# Nothing Made in Vain

**Published:** 1874-04

**Authors:** 


### NOTHING MADE IN VAIN.

All men and all things have their uses in the economy of creation; we may wonder sometimes what some men and women were made for. We look at a "weed," as we call it, and have regarded it for a lifetime as a worse than worthless thing, because it yields nothing, overspreads the ground, and great labor is expended in "rooting it out." The cotton seed on Southern plantations, within a short lifetime, was burned up to get it out of the way, but an oil is now pressed from it which is of great practical value; and, when the oil is pressed out of it, the refuse even is valuable as food for domestic animals. Formerly the Osage orange shrub had been considered useless except for hedges; now it is found to possess very valuable properties. The wood when boiled yields a beautiful, bright and permanent yellow dye; hides are tanned quicker with the wood than with oak bark, while the seeds yield a mild, limpid oil, which may be used as a substitute for sweet oil.

